# Emergency Department Comprehensive Social Risk Screening and Resource Referral Program

**DOI:** 10.5811/westjem.18578

**Published:** 2025-02-25

**Authors:** Kaytlena Stillman, Alex Dahut, Antonina Caudill, Katie Hren, Krystal Green, Marie Lauzon, Susan Jackman, Alexander Lawton, Tananshi Chopra, Joel Geiderman, Sam Torbati

**Affiliations:** * Cedars-Sinai Medical Center, Department of Emergency Medicine, Los Angeles, California; † Cedars-Sinai Medical Center, Office of Health Equity, Los Angeles, California; ‡ Cedars-Sinai Medical Center, Samuel Oschin Comprehensive Cancer Institute, Los Angeles, California

## Abstract

**Introduction:**

The emergency department (ED) is an appropriate location to screen for and address social risks among patients; however, a standardized process does not currently exist. Our objective in this study was to describe the implementation and findings of a social risk screening and resource referral program using a comprehensive screening questionnaire.

**Methods:**

We conducted a prospective, cohort study between July 2022–April 2023 at a single academic, urban ED in Los Angeles, CA. Trained staff on rotating shifts recruited ED patients between 6 am to midnight, with an average of 40 hours of coverage per week including weekends. Patients were excluded if they were <18 years of age, could not provide informed consent, or were deemed too medically unstable. Trained staff screened eligible consenting patients at ED bedside for social risks within 12 different domains of social determinants of health using a 19-question survey. Personalized resources were provided through an online platform or through direct communication with a social worker. Demographic data and patient responses were recorded in a deidentified database. We used a univariate logistic regression analysis to evaluate associations between demographic information and burden of social risk.

**Results:**

A total of 4,277 ED patients were considered for screening, and 1,677 (39.2%) were eligible: 1,473 (87.8%) patients consented to social risk screening, and 1,078 (73.2%) of them had at least one social risk as indicated by the screening questionnaire. The most commonly reported social risks were social isolation (39%) and depression (23%). Between 88.9–96.8% of patients categorized as medium social risk were successfully provided resources through the online platform. Between 80.8–100% of patients categorized into high social risk had successfully connected with a social worker while in the ED. In this sample, there were significantly higher odds of having greater than one social risk for female (odds ratio [OR] 1.30, 95% confidence interval [CI] 1.02–1.67) and Black patients (OR 1.37, 95% CI 1.02–1.85) compared to male and White patients, respectively.

**Conclusion:**

This study describes the findings from a comprehensive social risk screening and resource referral program at a large, urban, academic ED. The results will inform resource prioritization at the study institution. This model can serve as a basis for similar institutions to use, while individualizing their own approach.

## INTRODUCTION

The emergency department (ED) is a medical safety net for sociodemographically underserved populations.^1^ Because many ED patients have significant social needs, it is important that emergency clinicians be able to recognize and help address the upstream social and systemic factors that may have contributed to these patients’ ED presentations.^1^–^11^ Social risk is the term used to describe these specific adverse social conditions that lead to poor health.^12^,^13^ To assist ED recognition and mitigation of social risks, there has been a growing body of literature on the design and implementation of ED social risk screening.^3^,^14^–^24^ Institutions nationwide have developed their own social risk screening and resource referral programs, with many in the literature citing the use of a pre-validated screening tool for select populations only (eg, Medicaid patients) followed by referral to community-based resources.^19^,^21^,^23^ Some institutions have developed ED-based medical-legal partnerships to help address patient social needs.^25^ Despite these innovative practices, a standardized process does not currently exist, likely due to the variability in regional prevalence of social risk and institutional resources.^19^,^21^,^25^–^28^

Our objective in this study was to describe the implementation and findings of a social risk screening and resource referral pilot program at an urban, academic medical center’s ED. The unique screening questionnaire used in this study was developed at the study institution by an executive leadership steering committee with representation from all service lines across the health system. Questions were selected based on gaps in existing screening workflows, validated tools in the literature, needs specific to the community the hospital serves, federal mandates, and ease of patient understanding. The screening tool had already served as standard of care at several other access points in the hospital and was proposed for use in the ED to bridge the social needs gap of the health system where it is most needed. Unlike many other programs, this screening program was designed to capture as many different patient populations within the ED as possible, regardless of sociodemographic. While previous studies have evaluated screening tools with up to 10 different social risk domains (most using much fewer domains), in this study we also sought to evaluate more comprehensive social risk data than has previously been described in the literature by using a single screening questionnaire covering 12 different domains of social determinants of health.^19^,^21^,^25^–^28^

## METHODS

### Study Design and Setting

**Figure 1:**
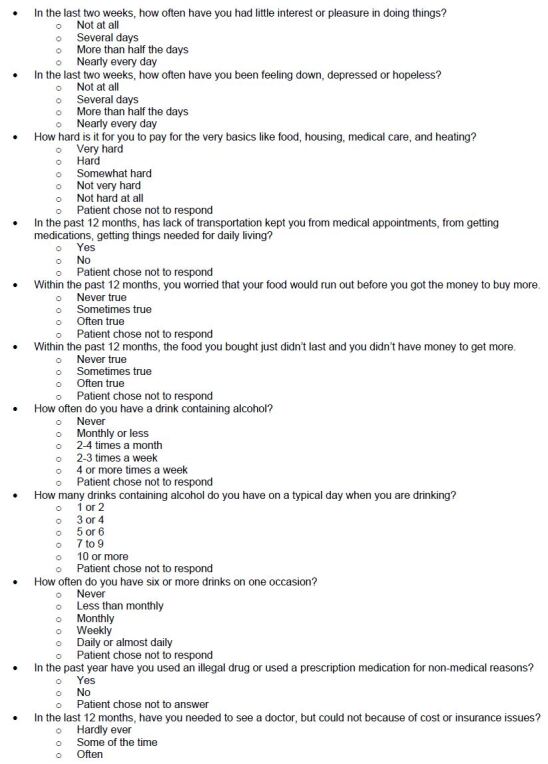
Social risk screening tool.

This was a prospective cohort study conducted between July 2022–April 2023 in an ED at a single academic, urban, quaternary medical center in Los Angeles, CA. The study was approved by the center’s institutional review board. Nineteen questions comprise the social risk screener and cover 12 domains of social determinants of health. Each individual question had been separately validated in previous studies to screen for its intended social risk within that domain (Table 1 and Figure 1).

**Population Health Research Capsule**

What do we already know about this issue?

*Many institutions nationwide have implemented varying forms of social risk screening and resource referral programs to promote health equity.*

What was the research question?

*What were the findings from a social risk screening program which used a more comprehensive screening tool?*

What was the major finding of the study?

*Among patients screened, 73% had at least one social risk with the most common being social isolation (39%) and depression (23%).*

How does this improve population health?

*By describing the implementation of this comprehensive social risk screening program, other institutions may utilize these tools in their own practice toward promoting health equity.*

### Selection of Participants

Trained research associates (RA) performed the bedside screening of ED patients. The RAs assessed patients for eligibility during scheduled shifts between 6 am and midnight, which provided 30–80 hours of coverage per week, including weekends. During the first month pilot of the screening process, we excluded patients who did not speak English and those who were in hallway beds. After this pilot, these patients were then included. A Martti translator (UpHealth Inc, Delray Beach, FL) assisted communication via iPad with patients whose native language was not English. Exclusion criteria were primarily age <18, appearing agitated or unstable, receiving active medical treatment, or having a primary psychiatric complaint as these patients are already screened for social risks by a dedicated psychiatric social worker at the study ED (Table 2).

### Interventions

**Figure 2:**
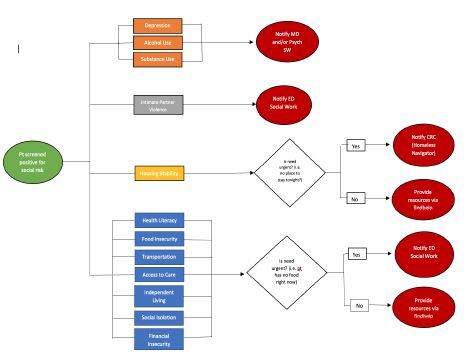
Social risk screening and referral workflow. *ED*, emergency department; *CRC*, community resource coordinator,

During working hours, a RA pre-screened current ED patients for eligibility. To assess exclusion criteria, the RA would first review a read-only version of the patient’s ED chart to obtain demographic information, primary chief complaint, emergency severity index (ESI), disposition, and “break the glass”/“research opt out” status. The rest of the exclusion criteria were assessed through communication with the medical team about the patient’s medical stability and whether any active bedside interventions were ongoing. All demographic information of these pre-screened patients were recorded in a de-identified REDCap (Reearch Electronic Data Capture) database, hosted at Cedars-Sinai Medical Center. The RA then approached eligible patients for consent and subsequently asked the screening questions at bedside. The social risk screener took an average of approximately five minutes to answer. The patient’s answers were recorded in the de-identified database. Positive social risks were stratified into moderate or high risk based on the patient’s answers to the individual pre-validated screening tools that comprise the comprehensive screener. Workflows were developed such that high levels of risks often triggered a social work referral, while moderate levels of risk were addressed with referral to available community-based resources (Figure 2).

Resource referrals were made using an electronic platform provided by a third-party vendor, findhelp (Aunt Bertha, Austin, TX).^29^ Through this platform, the RA entered the patient’s ZIP code and selected local resources pertaining to the patient’s social risks identified by the screener. The patient then received a printout, text message, or an email with these resources.

### Measurements and Analysis

Demographic characteristics were recorded for all patients in this sample. Other patient-level measurements were number of social risks present (if any), category of social risk, degree of risk (medium vs high), and whether resources were provided by the RA through the online platform or by communication with a social worker. We compared demographic characteristics between the total pre-screened population and the screened sub-population. Demographics associated with screening positive for at least one social risk and for greater than one social risk compared to no social risks were assessed by univariate logistic regression analysis. We reported odds ratios (OR) and their 95% confidence intervals (CI). A two-sided 0.05 significance level was used throughout. We made calculations using VassarStats, available online at http://vassarstats.net/.

## RESULTS

A total of 4,277 ED patients were pre-screened by RAs over the study period. Of these patients, 1,677 (39.2%) were eligible for social risk screening. The most common reason for exclusion (62.3% of excluded patients) was that the patient was in acute distress, ill-appearing, or agitated (see Table 2). Among the eligible patients, 1,487 (88.7%) consented to undergoing social risk screening, and ultimately 1,473 (99.1% of consented patients) were successfully screened.

The demographic features of the pre-screened patients and the screened patients were similar in terms of sex, race, and ethnicity. However, pre-screened patients were older on average than screened patients (Table 3). Among screened patients, 1,078 (73.2%) had at least one social risk as indicated by the screening questionnaire. The most commonly reported social risks were social isolation (39%) and depression (23%). Least frequently reported social risks were intimate partner violence (1%) and drug use (5%). Between 88.9–96.8% of patients categorized as medium social risk were successfully provided resources through the online platform. Between 80.8–100% of patients categorized into high social risk had successfully connected with a social worker while in the ED. Screened patients with social risks who did not receive resources or speak to a social worker where indicated had declined to engage in this option (Table 4). In the univariate logistic regression analysis (Table 5), we found that there were significantly higher odds of having greater than one social risk vs no social risks for female (OR 1.30, 95% CI 1.02–1.67) and Black patients (OR 1.37, 95% CI 1.02–1.85) compared to male and White patients, respectively.

## DISCUSSION

The goal of this study was to describe the implementation of a social risk screening and resource referral program in an ED at a large, urban, academic institution. A standardized program for social risk screening does not currently exist in large part due to the variability in needs of local patient populations and resource barriers to implementation that exist among regions and institutions. Therefore, it is crucial for each institution to assess its own capacity to screen for and address social risks among its ED patients and develop a program individualized to their needs and abilities. The ED involved in this study is fortunate to have several resources at its disposal including social workers, homeless navigators, clinical RAs supported by internal funding, and an online referral platform with thousands of resources available for patients within its catchment area. The program designed and piloted in this study enabled this ED to maximally harness its resources while creating little to no disruption in the ED workflows. The high percentages of patients with social risks who were appropriately connected with resources or with a social worker suggest that patients are generally receptive to resource referral and may have a high proportion of social needs in our population.

The demographic data collected for this study demonstrated that the patients who were eligible for screening for social risks were collectively a representative sample in terms of sex, race, and ethnicity of the larger ED patient population. However, patients screened for social risks were younger on average than those of the total pre-screened population. This is likely explained by the exclusion criteria of the study, encompassing patients who are ill-appearing, agitated, or lacking capacity to consent. Elderly patients are more susceptible to delirium and dementia, thus precluding them from social risk screening. However, it is worth noting that this institution also has access to a geriatric care coordinator who assists these patients with social needs outside the social risk screening program.

A major advantage of social risk screening is that it can inform investment in resources. Based on the findings from this screening program, the study ED plans to strengthen its availability of psychiatric social work services and has also educated the department’s clinicians about mental health resources for patients. The results of this study also identified female patients and Black patients as being populations vulnerable to an increased burden of social risk, and further work at the study institution should focus on identifying strategies to better address the needs of these ED patients. Knowing and addressing these needs is important not only so we may better understand the physiologic reasons for and treatment of disease in the ED, but also to help reduce health disparities among vulnerable populations in our region.^32^

The success and sustainability of a social risk screening program depends largely on its acceptability and accessibility to ED patients.^15^–^17^,^26^,^28^,^30^,^31^ Therefore, future directions for the program will include 30-day follow-up with patients who were screened and referred to resources to assess their satisfaction with the process, and to evaluate the success of community resource connections. The program will also be qualitatively studied to assess patient acceptability of the screening process. This is important because many questions related to assessing social risk are potentially stigmatizing, and screening for social risks is only useful when patients feel safe and comfortable answering. For example, this study found a low prevalence of reported intimate partner violence, but it is unclear whether this was due to the fact that intimate partner violence is a highly stigmatizing topic.

## LIMITATIONS

Limitations to interpretation of this study’s results are in large part due to the exclusion criteria that were applied and the resulting potential for selection bias. These criteria were imposed to maintain patient autonomy, research staff safety, minimize system redundancies, and work within resource availability. For example, patients with primary psychiatric complaints were excluded because these patients are already thoroughly screened for social risks by a dedicated psychiatric social worker whose position already existed at this institution. Due to the exclusions, the results of this study are subject to potential selection bias, especially with regard to the medical team’s interpretation of whether a patient was medically stable enough to undergo screening. However, because this program was a pilot the exclusion criteria were necessary to create a feasible model that could be continued within the study institution’s resource constrictions while creating minimal disturbance to clinical workflows. The COVID-19 exclusion was in place due to epidemiologic recommendations around contact precautions and resource maximization at the time the pilot was conducted, but it will be relaxed moving forward. Additionally, we recognize that social risks for an individual are often dynamic; so, future plans are to screen every eligible patient at each ED encounter, regardless of their previous screening status.

Patients in this sample were limited to those who were present during research staff working hours. Also, during the first month of enrollment, non-English speaking and hallway patients were excluded to assess process feasibility. This initial exclusion may have impacted the demographic makeup of our screened sample; however, it was unlikely to have had a major effect given the very small sample size recruited in the first month. Another limitation of the study is that demographic information was collected from chart reviews. At the study institution, demographic data is self-reported and documented by registration staff; however, errors of input are possible. Additionally, we do not have available data on why 190 (11.3%) eligible patients declined consent for screening. We also do not know why 14 (0.9%) consented patients did not ultimately receive screening; however, anecdotal reports from RAs indicate that these consenting patients were likely discharged or transferred to an inpatient room before screening could be performed.

## CONCLUSION

This study describes the findings from a social risk screening and resource referral program at a large, urban, academic ED. The screening tool used in this study is more comprehensive than has previously been described in the literature. The results will inform future directions in terms of resource prioritization. This model can serve as a basis for similar institutions to use, while individualizing their own approach. The next steps for this program will be to study patient acceptability of social risk screening and 30-day follow-up of resource utilization.

## Figures and Tables

**Table 1:** Social determinant of health domains and the pre-validated tools from which screening questions are derived.

Domain	Pre-validated assessment tool	# of questions
Depression	PHQ-2	2
Financial Resource Strain	Accountable Health Communities Health-related Social Needs Screening Tool	1
Food Insecurity	Hunger Vital Signs	2
Housing Stability	PRAPARE	2
Transportation Needs	Modified PRAPARE (Epic)	1
Access to Care	Health Leads (modified)	1
Health Literacy	Short Test of Functional Health Literacy in Adults	1
Independent Living	CDC Behavioral Risk Factor Surveillance System	1
Intimate Partner Violence	Partner Violence Screen	1
Social Connections	UCLA 3-Item Scale	3
Alcohol Use	AUDIT-C	3
Substance Use	DAST-10	1
Total		19

*CDC*, US Centers for Disease Control and Prevention*; PHQ-2*, Patient Health Questionnaire-2; *PRAPARE*, Protocol for Assessing and Responding to Patients Assets, Risks, and Experiences; *AUDIT-C*, Alcohol Use Disorders dentificationn Test-Consumption; *DAST-10*, Drug Abuse Screening Test.

**Table 2:** Reasons for exclusion from screening (not mutually exclusive).

Exclusion criteria	Frequency (%) N=2,600
Pediatric patient <18 years old	29 (1.1)
Adults who lack capacity to consent	86 (3.3)
Non-English speaking patient (first month of pilot only)	210 (8.08)
Patient in acute distress, ill-appearing, or agitated	1,621 (62.3)
Patients with a primary psychiatric chief complaint	231 (8.9)
Patients receiving active nursing, PA, or MD assessment or intervention	164 (6.3)
Patient with suspected COVID-19 or other airborne disease	355 (13.7)
Patients who will be imminently discharged	482 (18.5)
Clinician does not believe participation would be in patient’s best interest	30 (1.2)
Patient previously participated in the same social risk screening study	28 (1.1)
Medical records flag “break the glass” or “research opt out”	84 (3.2)

*PA*, physician assistant; *MD*, doctor of medicine, *“break the glass*,” access restricted patient’s record in an emergency.

**Table 3:** Demographic information for pre-screened and screened patients.

Demographic variable	Pre-screened patients(%) N=4,277	Screened patients (%) N=1,473
Sex		
Male	2,080 (48.6)	724 (49.2)
Female	2,195 (51.3)	748 (50.8)
Unknown	2 (0.0)	1 (0.1)
Race		
White	2,623 (61.3)	890 (60.4)
Black	959 (22.4)	363 (24.6)
Asian	244 (5.7)	69 (4.7)
American Indian or Alaskan Native	26 (0.6)	9 (0.6)
Native Hawaiian or Other Pacific Islander	12 (0.3)	5 (0.3)
Other	413 (9.7)	137 (9.3)
Age		
18–24	261 (6.1)	117 (7.9)
25–34	635 (14.8)	261 (17.7)
35–44	644 (15.1)	245 (16.6)
45–54	574 (13.4)	202 (13.7)
55–64	630 (14.7)	222 (15.1)
≥65	1,519 (35.5)	426 (28.9)
Unknown	14 (0.3)	0 (0.0)
Ethnicity		
Hispanic	762 (17.8)	247 (16.8)
Not Hispanic	3,437 (80.4)	1,202 (81.6)
Prefer not to answer	78 (1.8)	24 (1.6)

**Table 4:** Social risks of patients screened in the emergency department and resources provided.

Social risk	Frequency of social risk (%) (N=1,473)	Local resource referrals (% of those at risk)	Successful connections with social worker (% of those at high risk)
Social connections			
High risk	565 (38.3)	539 (95.4)	-
Independent living			
High risk	222 (15.1)	207 (93.2)	-
Transportation needs			
High risk	120 (8.1)	114 (95.0)	-
Health literacy			
High risk	117 (7.9)	104 (88.9)	-
Housing stability			
Moderate risk	145 (9.8)	139 (95.9)	-
High risk	19 (1.3)	-	16 (84.2)
Intimate partner violence			
High risk	16 (1.1)		16 (100.0)
Alcohol use			
High risk	155 (10.5)	-	#
Depression			
High risk	338 (22.9)	-	#
Drug use			
High risk	79 (5.4)	-	#
			#467 (81.6)
Financial strain			
Moderate risk	272 (18.4)	262 (96.3)	-
High risk	233 (15.8)	-	*
Food insecurity			
Moderate risk	217 (14.7)	210 (96.8)	-
High risk	18 (1.2)	-	*
Access to care			
Moderate risk	134 (9.1)	127 (94.8)	-
High risk	67 (4.5)	-	*
			*257 (80.8)

# Combined connections to social work for alcohol use, depression, and drug use.

* Combined connections to social work for high-risk financial strain, food insecurity, and access to care.

**Table 5:** Demographic characteristics and odds ratios of social risks.

Demographic variable	No social risks (%) n=395	At least 1 social risk (%) n=1,078	OR for at least 1 vs no social risks (95% CI)	>1 social risks (%) N=699	OR for >1 vs no social risks (95% CI)
Sex					
Male	205 (51.9)	519 (48.1)	Reference	316 (45.2)	Reference
Female	190 (48.1)	558 (51.8)	1.16 (0.92–1.46)	382 (54.6)	1.30 (1.02–1.67)
Unknown	0	1 (0.1)	*	1 (0.1)	*
Race					
White	242 (61.3)	648 (60.1)	Reference	400 (57.2)	Reference
Black	86 (21.8)	277 (25.7)	1.20 (0.91–1.60)	195 (27.9)	1.37 (1.02–1.85)
Asian	26 (6.6)	43 (4.0)	0.62 (0.37–1.03)	25 (3.6)	0.58 (0.33–1.03)
American Indian or Alaskan Native	2 (0.5)	7 (0.6)	*	7 (1.0)	*
Native Hawaiian or other Pacific Islander	2 (0.5)	3 (0.3)	*	3 (0.4)	*
Other	37 (9.4)	100 (9.3)	1.01 (0.67–1.51)	69 (9.9)	1.13 (0.73–1.73)
Age					
18–24	30 (7.6)	87 (8.1)	Reference	54 (7.7)	Reference
25–34	64 (16.2)	197 (18.3)	1.06 (0.64–1.75)	142 (20.3)	1.23 (0.72–2.10)
35–44	66 (16.7)	179 (16.7)	0.94 (0.57–1.54)	115 (16.5)	0.97 (0.56–1.66)
45–54	49 (12.4)	153 (14.2)	1.08 (0.64–1.82)	102 (14.6)	1.16 (0.66–2.03)
55–64	59 (14.9)	163 (15.1)	0.95 (0.57–1.59)	110 (15.7)	1.04 (0.60–1.79)
≥65	127 (32.1)	299 (27.7)	0.81 (0.51–1.29)	176 (25.2)	0.77 (0.47–1.27)
Ethnicity					
Hispanic	72 (18.2)	175 (16.2)	Reference	117 (16.7)	Reference
Not Hispanic	318 (80.5)	884 (82.0)	1.14 (0.84–1.55)	571 (81.7)	1.11 (0.80–1.53)
Prefer not to answer	5 (1.3)	19 (1.8)	*	11 (1.6)	*

* Frequency too small.

*OR*, odds ratio; *CI*, confidence interval.
